# Mathematical Modeling: A Tool for Optimization of Lipid Nanoparticle-Mediated Delivery of siRNA

**DOI:** 10.1016/j.omtn.2017.04.003

**Published:** 2017-04-12

**Authors:** Radu Mihaila, Dipali Ruhela, Edward Keough, Elena Cherkaev, Silvia Chang, Beverly Galinski, René Bartz, Duncan Brown, Bonnie Howell, James J. Cunningham

**Affiliations:** 1 Sirna Therapeutics, 1700 Owens Street, Fourth Floor, San Francisco, CA 94158, USA; 2 Department of RNA Therapeutics, Merck Sharp & Dohme Corp., West Point, PA 19486, USA; 3 Department of Mathematics, University of Utah, Salt Lake City, UT 84112, USA

**Keywords:** RNA interference, siRNA, lipid nanoparticles, mathematical modeling, intracellular trafficking, cellular pharmacokinetics

## Abstract

Lipid nanoparticles (LNPs) have been used to successfully deliver small interfering RNAs (siRNAs) to target cells in both preclinical and clinical studies and currently are the leading systems for in vivo delivery. Here, we propose the use of an ordinary differential equation (ODE)-based model as a tool for optimizing LNP-mediated delivery of siRNAs. As a first step, we have used a combination of experimental and computational approaches to develop and validate a mathematical model that captures the critical features for efficient siRNA-LNP delivery in vitro. This model accurately predicts mRNA knockdown resulting from novel combinations of siRNAs and LNPs in vitro. As demonstrated, this model can be effectively used as a screening tool to select the most efficacious LNPs, which can then further be evaluated in vivo. The model serves as a starting point for the future development of next generation models capable of capturing the additional complexity of in vivo delivery.

## Introduction

The RNA interference (RNAi) pathway is a naturally occurring gene-silencing mechanism in many eukaryotes that controls the expression of endogenous genes.^1^ This pathway can be exploited to silence target genes, in both cells and animals, with synthetic small interfering RNA (siRNA) duplexes, typically 21–23 base pairs in length.^2^ In order to specifically silence the expression of genes by RNAi, siRNA must be delivered across the cell membrane into the cytoplasm, where the passenger strand of the duplex is cleaved and released. Only the guide strand is loaded into a protein complex, called the RNA induced silencing complex (RISC), which mediates target mRNA cleavage. Argonaute 2 (Ago2) is one of the main components of RISC, which participates in the binding/catalysis event.^3^ Specific degradation of target mRNAs ultimately results in a reduction of the encoded target proteins over time. This technology is not only a valuable tool to study protein function in vivo and in vitro (e.g., for target validation), but also has potential for use as a new class of therapeutic agents.

The successful delivery of siRNA into the cytosol remains a major challenge and, in particular, endosomal escape of the siRNA into the cytoplasm is believed to be a key step.^4^, ^5^ A variety of strategies to improve siRNA uptake into cells have been reviewed,^6^ which include the packaging of siRNA into lipid nanoparticles (LNPs). LNP is a type of cationic liposomal drug carrier that serves as an effective delivery vehicle for siRNA.^7^, ^8^ It can be composed of several components, such as cationic lipids, helper lipids, and PEG (polyethylene glycol) conjugates in specific molar ratios. When incorporated into LNPs, fusogenic cationic lipids are hypothesized to facilitate cellular uptake and endosomal escape by interacting with the cell membrane and/or may help destabilize endosomal membranes to induce unpackaging of the cargo into the cytosol.^4^, ^9^, ^10^ LNPs have recently been shown to successfully deliver siRNA in preclinical and clinical trials.^7^, ^11^, ^12^, ^13^, ^14^, ^15^, ^16^ Given the large number of cellular factors that can impact the RNAi delivery pathway, integrated mathematical modeling using differential equations is a viable approach to quantify the complex relationships among cellular barriers and delivery vector modifications.^17^ Other groups have built large-scale computational models for RNAi.^18^, ^19^ One of the obstacles in the implementation of such models, to optimize the delivery platforms for siRNA, is that experimental biochemists are not typically equipped with computational expertise sophisticated enough to generate quantitative predictions from the models. Furthermore, the larger the number of parameters in a model, the accuracy of the model predictions suffers because a much larger number of experiments is needed in order to accurately determine the model parameters. Often, the modeler chooses to fit the unknown parameters, and the larger the number of parameters one chooses to fit, the less quantitative the model becomes. Undoubtedly, the already published Davis approach^18^ is elegant but is more qualitative than quantitative in nature due to the large number of fitted parameters (17 out of 29 parameters were fitted).

**Figure 1:**
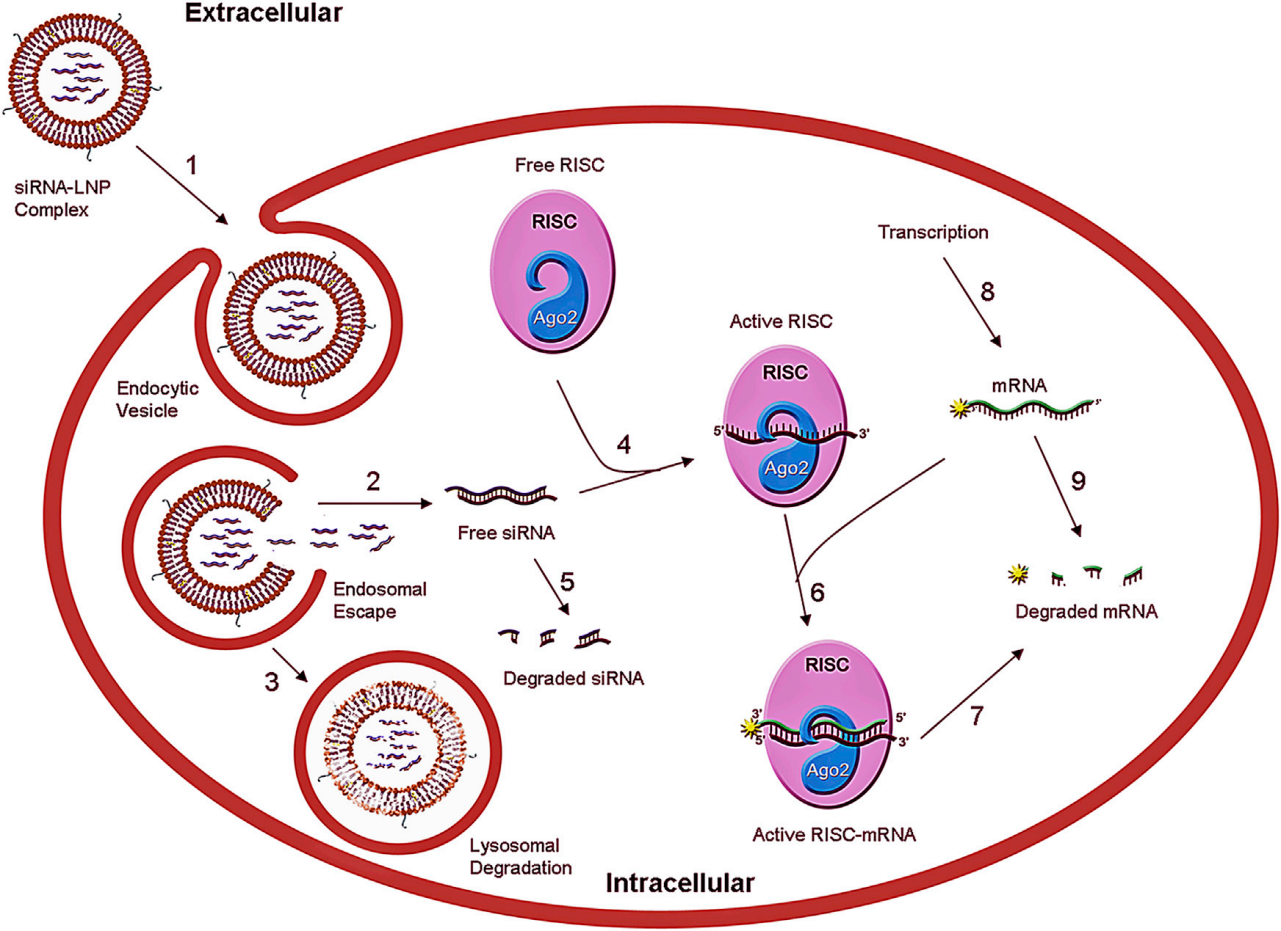
Kinetic Model for LNP-Mediated siRNA Delivery Schematic of the kinetic model with the key steps involved in the lipid-nanoparticle-mediated siRNA delivery into the hepatocytes, resulting in gene silencing. The various steps involved are as follows: (1) LNP crossing the plasma membrane; (2) endosomal escape/unpackaging; (3) lysosomal degradation; (4) siRNA loading onto RISC; (5) degradation of siRNA in the cytoplasm; (6) formation of active RISC with target mRNA; (7) cleavage of target mRNA by RISC; (8) transcription rate (of mRNA); and (9) degradation of mRNA.

The major goal of this work is the design and validation of a simplified yet predictive mathematical model that can compare the relative kinetics of different classes of LNPs in vitro, without having to carry out actual biochemical experiments. We essentially modeled the critical steps involved in delivery of siRNA to cells in vitro. We also tested our delivery vehicles in vivo, and the differences between the various siRNA-LNPs tested further illustrated the utility of this approach and validity of the model. Although still poorly understood, the critical steps of intracellular delivery have directly or indirectly been delineated using elegant imaging studies.^5^, ^20^ We have made an attempt to build a simplified mathematical model by capturing these critical steps (crossing plasma membrane, endosomal escape/unpackaging, loading of siRNA onto RISC, and mRNA knockdown) in our model. For ease of use and widespread applicability, models should be as simple as possible as long as they reproduce empirical findings; ideally, models should have fewer degrees of freedom than the training data.^21^ Our aim was to develop the simplest possible model (Figure 1) that captures the key features of LNP-mediated delivery of siRNAs, consistent with the level of available experimental data, and demonstrate its utility in screening various siRNAs/LNPs for target mRNA knockdown in vitro, without having to carry out a large number of conventional biochemical assays, which turns out to be expensive and tedious.

## Results

### Kinetic Model for Delivery of LNP-Mediated siRNAs

The extracellular and intracellular volumes are represented as separate modules in a two-compartment model (Figure 1). The various steps are numbered and described in the legend. The units of concentration for the species used in the model are number of molecules per cell. The model is composed of five ordinary differential equations (ODEs) describing the dynamics of the state variables. The input of the model is the extracellular LNP concentration (E), and mRNA (M) expression is the output. The steps are formulated as elementary unimolecular and bimolecular processes with r = k * [A] for a unimolecular reaction, where A indicates the total number of molecules per cell, r is a rate, and k is the rate constant. For a bimolecular reaction involving A and B, the rate is represented as r = k * [A][B]. Hill functions and other higher-order algebraic functions were not used because they represent approximations to cascades of elementary reactions. Thus, nonlinear behaviors arise in the model only from the interplay of simple reactions. The ODEs incorporated in our model are as follows:

(Equation 1) d[N]/dt=k1∗[E]-(k2+k3)∗[N]

(Equation 2) d[S]/dt=k2∗[N]-k5∗[S]-k4∗[S]∗[R]

(Equation 3) d[SR]/dt=k4∗[S]∗[R]-k6∗[M]∗[SR]

(Equation 4) d[SRM]/dt=k6∗[M]∗[SR]-k7∗[SRM]

(Equation 5) d[M]/dt=k8-k9∗[M]-k7∗[SRM].

The variables and their initial values used in the above equations are described in Table 1. The model contains nine parameters obtained from direct experimental measurements and published literature,^18^, ^22^, ^23^, ^24^, ^25^ as illustrated in Table 1. We modeled these differential equations in the Simbiology toolbox developed for MATLAB users (code is included in the Supplemental Information; The Mathworks).

**Table 1:** Description of the Various Model Variables and Model Parameters Used to Optimize the Model

Model Variables	Description	Initial Value (Molecules)	Reference
E	extracellular LNP	10 nM	a
N	endosomal LNP	0	
S	free sirna (cytoplasm)	0	
R	RISC complexes	10,000 copies per cell	^18^
SR	Ago2-bound siRNA	0	
SRM	active RISC mRNA	0	
M	mRNA	100 copies per cell	a
Extracellular compartment		3E-4 L	a
Intracellular compartment		1.4E-12 L	^22^

**Model Parameters (for LNP201)**	**Description**	**Value**	**Reference**

k1	LNP crossing the plasma membrane	0.005 L/hr	a
k2	endosomal escape/unpackaging	5.00E-04 L/hr	a
k3	lysosomal degradation	3 L/hr	^23^
k4	siRNA loading onto RISC	0.001 L/nM*hr	a
k5	degradation of siRNA in the cytoplasm	0.03 L/hr	^24^
k6	formation of active RISC with target mRNA	0.1 L/nM*hr	^25^
k7	cleavage of target mRNA by RISC	7.2 L/hr	^25^
K8	transcription rate of mrna	100 copies//hr	18, 38
k9	degradation of mRNA	1 L/hr	^39^
Time of escape, k2_RNAiMAX		5–20 min, 0.004	
Time of escape, k2_LNP05		1.5–2 hr, 0.002	
Time of escape, k2_LNP-(1,3)-diether		1–1.5 hr, 0.01	

a Values determined experimentally.

Equation 1 follows the concentration of LNP in the endosome (N) over time. The initial value for the extracellular LNP (E) species is derived from the experimental conditions used for LNP201 (as discussed later). The extracellular compartment feeds the LNP (E) into the endosomal pathway. The first constant, k1, represents the intracellular uptake parameter, and the second portion of the equation is represented by two degradation terms: the escape/unpackaging of the siRNA cargo out of the endosome (k2) and the siRNA cargo degradation term as the LNP travels to the lysosomal pathway (k3). Equations 2–4 represent the rates and constants involved in Ago2 (R) uptake of free siRNA (S) to form the complex (SR) and formation of the RISC-mRNA active complex (SRM) that undergoes target cleavage. Equation 5 follows the output M as it changes over time, depending on the transcription rate (k8), modeled with no feedback for the duration of the experiment (24 hr). The degradation term (k9) and cleavage rate (k7) impact the silencing outcome. Once unpackaged out of the delivery vehicle, the siRNA-binding/cleavage kinetics drives the potency. However, when we compare different classes of LNPs with the same cargo, the reaction kinetics is driven by the first two steps because k1, k2, and k3 are intrinsic characteristics of the vehicle and not the siRNA.

We have assumed a constant rate of transcription (k8), modeled without feedback, and used a degradation rate of the transcript mRNA that we considered to be accurate enough for the relatively short time span of the experiment. Because degradation of the siRNA cargo in the extracellular LNP is negligible, we excluded this parameter from our model.^26^ The initial value of mRNA was set at the steady-state value, which was inferred from internal expression data relative to a glyceraldehyde 3-phosphate dehydrogenase (GAPDH) control. The baseline values for the RISC species, formation of an active RISC-mRNA complex, and cleavage rates were based on literature values. The lysosomal degradation rate and siRNA cytoplasmic degradation rates were also based on literature values for liposomes and chemically modified siRNAs, as published previously.^24^ In order to minimize variability, the experimental conditions were kept constant between assays, and we purposefully limited all our in vitro experiments to 24 hr to minimize any cell-doubling effect and therefore no dilution parameter was required. Parameters were also appropriately adjusted for different cell lines, which enabled us to get experimental values for rate constants, irrespective of the cell line used.

### Experimental Determination of Rate Constants

First, in vitro experiments were conducted to gain insights into the actual kinetics of LNP-driven siRNA-mediated gene silencing. Because noninvasive monitoring of each individual kinetic step at a microscopic level is not possible, we developed a compartment model to correlate our microscopic observations with our kinetic biochemical data. Kinetic parameters for the model were derived from the experimental data obtained and the biochemical data described later. We partitioned the data into working data to which the three key parameters k1, k2, and k4 were fitted, and then we tested our modeling results against independently obtained test data in order to validate the model. The model’s explanatory power comes from being able to account for the data for which it was not fitted.

**Figure 2:**
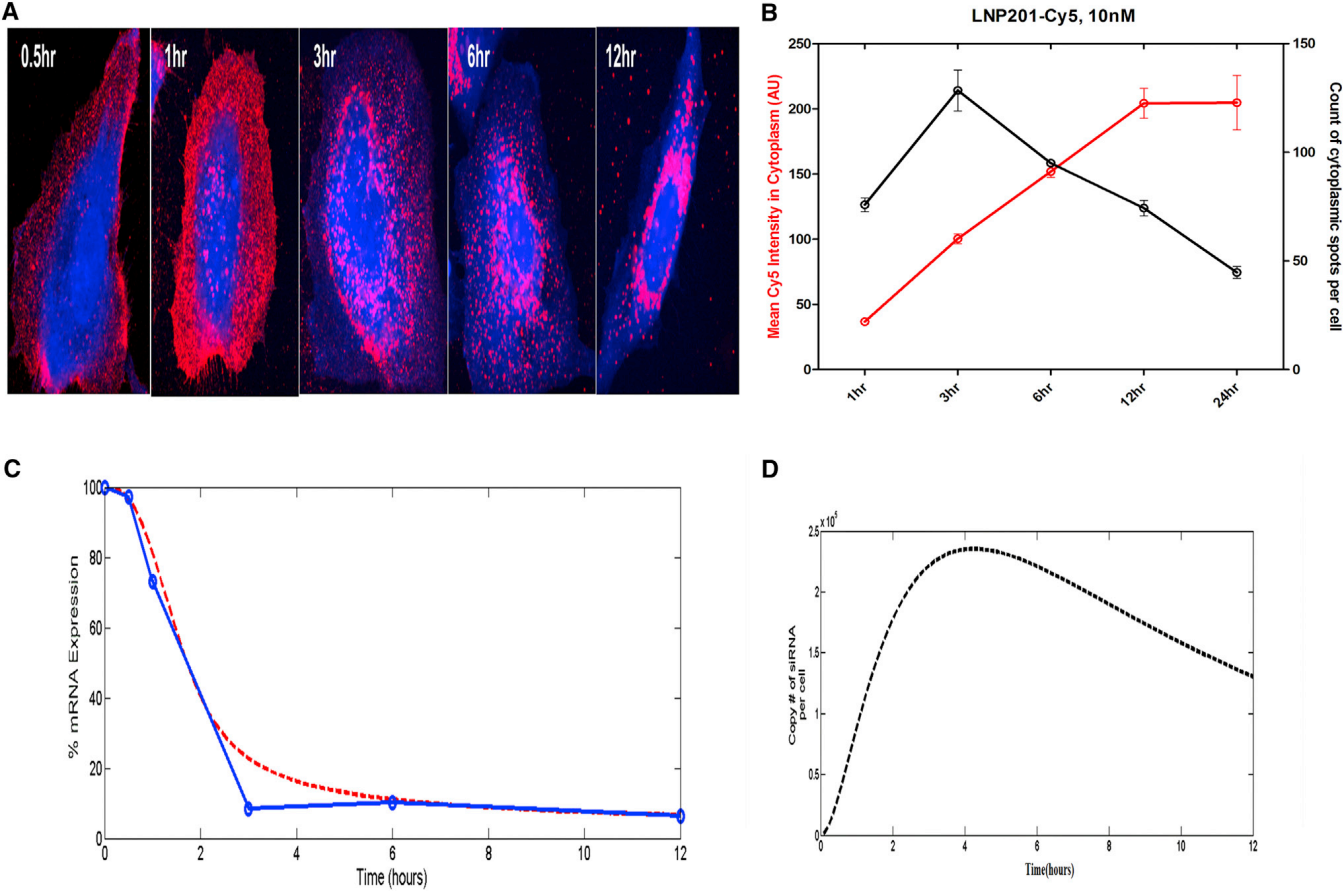
Analysis of Uptake of SSB-LNP201 In Vitro (A) 40X magnification images from the Opera confocal microscope showing uptake of 10 nM Cy5-labeled siRNA (red) encapsulated in LNP201 in Cell Tracker (blue) labeled HeLa cells from 0.5 to 12 hr. (B) Image analysis of the above Cy5-labeled siRNA-LNP201 complex. (C) Blue curve shows the in vitro SSB mRNA knock down after transfection of HeLa cells with 10 nM Cy5-labeled SSB siRNA encapsulated in LNP201, and these data were used to optimize the values of k1, k2, and k4. Red curve shows the SSB mRNA knock down after transfection, as predicted by the mathematical model using the thereby optimized in vitro parameters, as given in Table 1. (D) Model simulation of siRNA uptake that replicates the experimentally measured uptake of cell-associated Cy5-SSB-LNP201, as depicted in Figure 2B.

We used Cy5-labeled SSB (Sjogren syndrome antigen B) siRNA, encapsulated in LNP201,^27^ to obtain the very first set of experimental values to fit into our model. This siRNA (Figure S1) targets the ubiquitously expressed SSB gene.^28^ Figure 2A shows the confocal microscopic images tracking qualitative cellular uptake of the siRNA. Cell Tracker Blue was used as the cytoplasmic marker to identify the cell body and plasma membrane boundary. The pattern of Cy5-siRNA suggests that LNP201 complexes primarily localize to the plasma membrane at 0.5 hr, appear in endosomal compartments between 1 and 3 hr, and begin to appear in larger perinuclear organelles as well by 6 hr. Image analysis of the Cy5-labeled siRNA-LNP201 complex revealed that by 3 hr, the uptake into endosomes had peaked at >100 cytoplasmic spots identified per cell, and the overall cellular content of siRNA continued to increase over 12 hr, as measured by the Cy5 intensity in the cytoplasm (Figure 2B). We then experimentally measured the cell-associated Cy5-SSB-LNP201 complex uptake at a concentration of 10 nM. We realize that there are limitations to fluorophore-tracking experiments because there is a possibility that Cy5 fluorophore could be cleaved away from the siRNA. In parallel, we also ran a biochemical assay and analyzed target expression independently. The first two equations in our model are linear ODEs and can be solved analytically or numerically. This straightforward approach allowed us to determine the k1, k2, and k4 constants from the cell-associated siRNA uptake. Subsequently, we incorporated the above obtained values of k1, k2, and k4 into our model to determine mRNA expression with time upon SSB-LNP201 treatment and verified that our numerical results were a good fit when plotted against the actual experimental data (Figure 2C). The siRNA uptake was simulated using our compartment model, which correlated with the experimentally measured cell-associated Cy5-SSB-LNP201 (Figure 2D).

**Figure 3:**
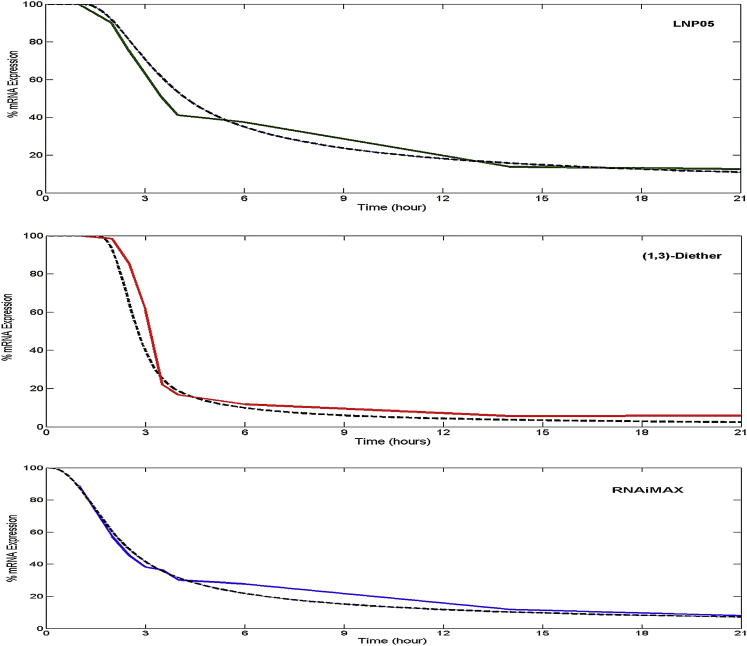
SSB mRNA Knockdown Kinetics In Vitro mRNA knockdown kinetics over 21 hr for 10 nM SSB using Hepa1-6 cells at a density of 10,000 cells per well for (A) LNP05 (green trace), (B) LNP(1,3)-diether (red trace), and (C) RNAiMAX (blue trace). Each data point is a mean of ten independent runs. The black curves in each plot illustrate corresponding model predictions after applying a time delay factor to k2 for the LNPs.

**Figure 4:**
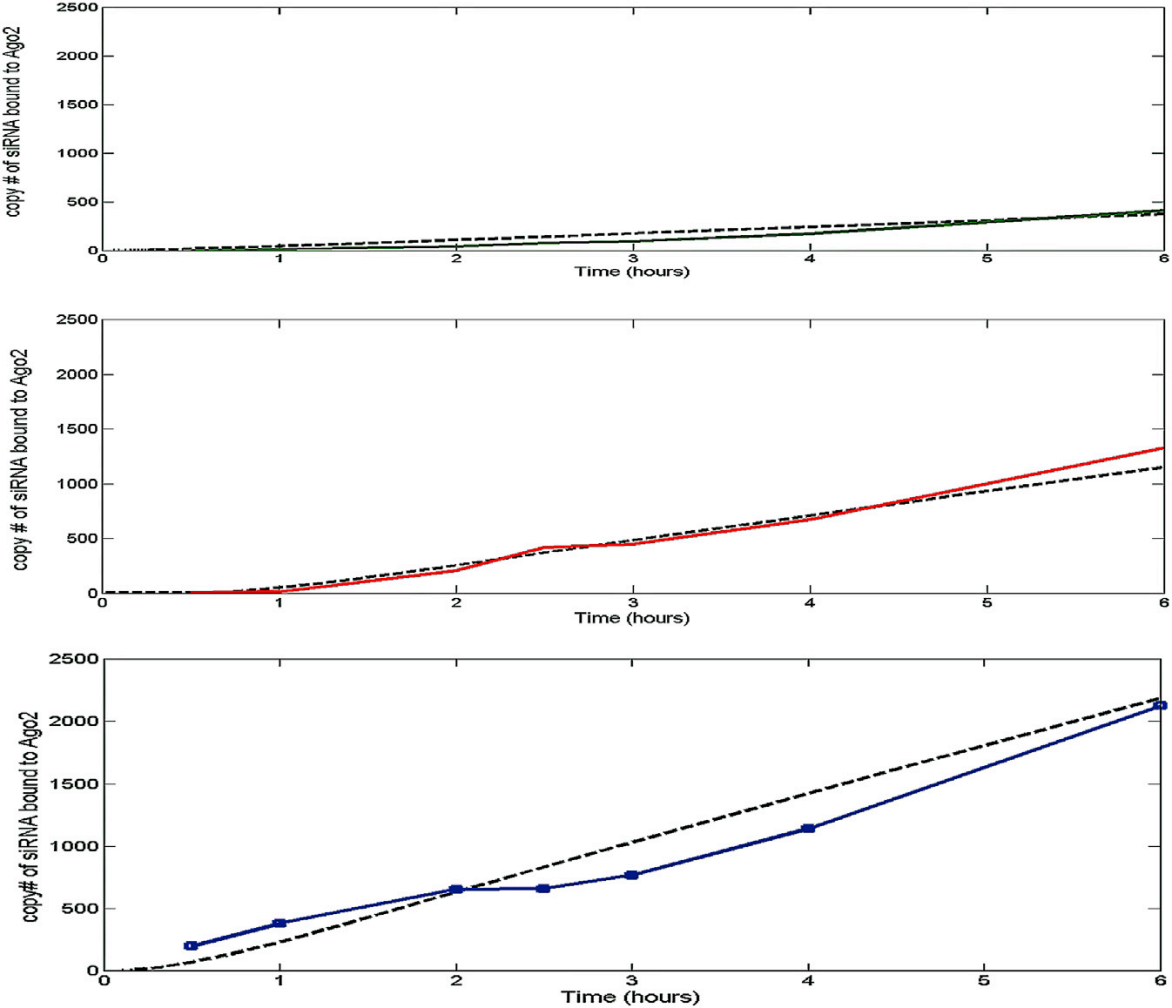
SSB Ago2-Binding Kinetics In Vitro Ago2-binding kinetics of SSB complexed with (A) LNP05 (green trace), (B) LNP(1,3)-diether (red trace), and (C) RNAiMAX (blue trace). The assay was done with Hepa 1-6 cells at a density of 4.4 × 10^6^/10-cm plate for a time period of 6 hr post transfection. Kinetics of Ago2 binding evidently varies from one delivery vehicle to another. The black curves in each plot show the kinetics of Ago2 binding for SSB, with the corresponding delivery vehicles as predicted from the mathematical model using the baseline values illustrated in Table 1.

SSB siRNA complexed with RNAiMAX (a widely used transfection reagent) and two different LNPs (LNP05 and LNP-(1,3)-diether) were then assessed for SSB mRNA knock down with time. There was an evident shift in kinetics for the three different vehicles at 3.5 hr. We first refined k1 and k2 for SSB-RNAiMAX, keeping the other parameters constant (same as L201), to fit the experimental data (Figure 3C) that we obtained. Subsequently, for LNP05 and LNP(1,3)-diether, it was necessary to adjust k2 to account for the unexpected initial delay that we observed with LNPs (and for this, we incorporated the Heaviside function). After this exercise, the simulations matched the actual in vitro data for the two delivery vehicles (Figures 3A and 3B). Then, an Ago2-binding assay was carried out in vitro with the SSB siRNA complexed with RNAiMAX, LNP(1,3)-diether, and LNP05 respectively over 6 hr (Figure 4). RNAiMAX showed the fastest Ago2-binding kinetics (Figure 4C), followed by LNP(1,3)-diether (Figure 4B) and LNP05, respectively (Figure 4A). To our satisfaction, we were able to simulate the actual in vitro Ago2-binding kinetics for the three delivery vehicles using our model after incorporating the optimized parameters as obtained above, as depicted by black dotted lines, further validating the model’s accuracy.

**Figure 5:**
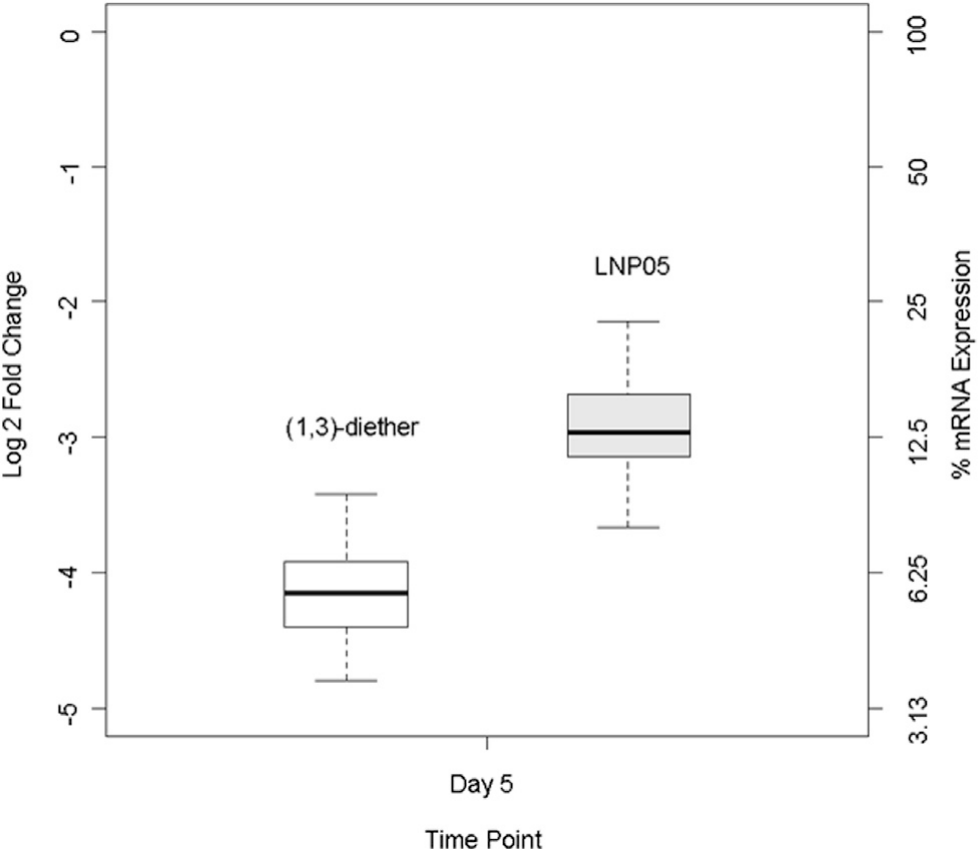
SSB Gene Silencing Kinetics In Vivo Evaluation of SSB gene silencing in vivo using LNP05 and LNP(1,3)-diether on day 5. C57BL/6 mice were intravenously dosed at 3 mg/kg with SSB siRNA encapsulated within LNP(1,3)-diether and LNP05 delivery vehicles, respectively. The livers were collected 5 days post-dose, and the knockdown of the SSB mRNA was determined relative to GAPDH expression. The log 2-fold change is relative to the PBS-treated animals.

To assess if our quantitative in vitro prediction correlated with in vivo results, a historical in house dataset of rodent studies was mined to compare the performance of LNP05 to LNP(1,3)-diether in knocking down the SSB gene, a standard positive control used in all in-house in vivo experiments. A total of 111 mice received either the LNP05 (71 mice) or LNP(1,3)-diether (40 mice) SSB treatments. LNP(1,3)-diether significantly and consistently outperformed LNP05 in the dataset, achieving a more than 2-fold improvement, with a p value < 10–16 by the Mann-Whitney nonparametric test (Figure 5).

### Sensitivity Analysis of the Model Parameters and Optimization Strategy

**Figure 6:**
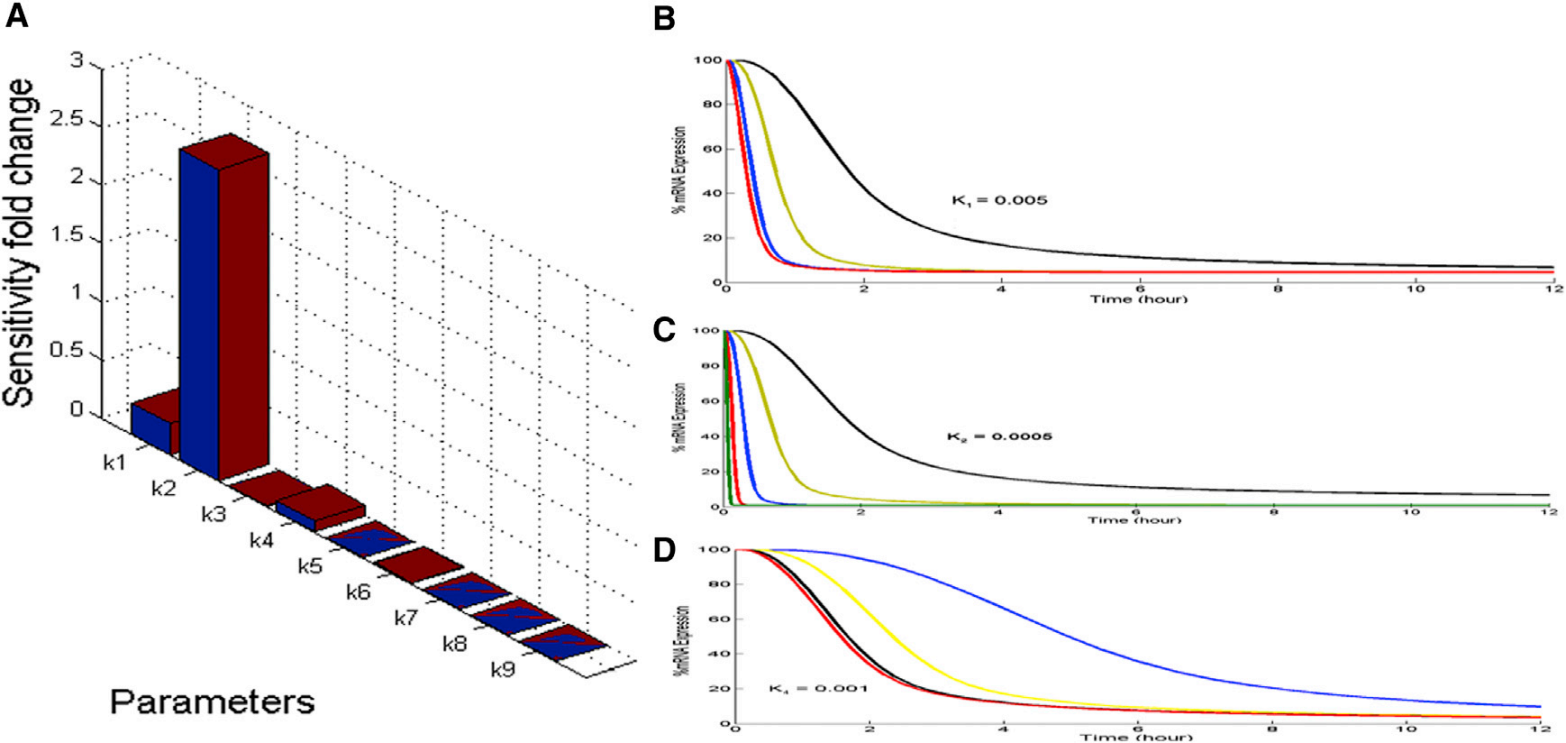
Sensitivity Analysis of the Model Parameters (A) Sensitivity analysis carried out on all the model parameters using the Simbiology toolbox in MATLAB. Modeling the mRNA knockdown with time by (B), varying k1 and keeping the other variables constant at their baseline values as illustrated in Table 1; k1 was varied 10^3^-fold starting from 0.1 (baseline value) and going up to 1,000. (C) Varying k2 and keeping the other variables constant at their baseline values as illustrated in Table 1; k2 was varied 10^6^-fold starting from 0.0001 (baseline value) and going up to 500. (D) Varying k4 and keeping the other variables constant at their baseline values as illustrated in Table 1; k4 was varied ∼10^4^-fold starting from 0.001 (baseline value) and going up to 10. The software computes local sensitivities by combining the original ODE system for a model with the auxiliary differential equations for the sensitivities. The additional equations are derivatives of the original equations with respect to parameters. Time-dependent derivatives (∂M/∂k1-9), where the numerator is the sensitivity output of mRNA silencing and the denominators are the sensitivity inputs to sensitivity analysis.

Sensitivity analysis was carried out to determine the relative impact of each parameter on the model output (Figure 6A). From our analysis, the endosomal escape parameter (k2) turned out to be by far the most sensitive parameter, followed by significant contribution from k1 to k4. In order to further evaluate the significance of k2 on mRNA expression relative to k1 and k4, we carried out parameter scans by varying these three variables one at a time, keeping all the other parameters constant at their baseline values, as illustrated in Table 1. As depicted in Figures 6B and 6D, we hit a barrier in activity for k1 and k4. The parameter scans for k1, k2, and k4 over five orders from the corresponding baseline values suggested that optimization of k2 will lead to the best improvement in silencing activity (Figure 6C). The goal of the present work is the rational design of an optimal siRNA delivery strategy. Our overall strategy is deceptively simple: find the slow step in a family of delivery vehicles and design more optimal delivery vehicles with better release kinetics.

### Prediction of Knockdown with Novel Combinations of siRNAs and LNP-Delivery Vehicles

Furthermore, we performed an actual in vitro kinetic assay (mRNA knockdown versus time) for a different siRNA targeting CBR4 (NM_145595) (a carbonyl reductase enzyme and a potential therapeutic target). Two distinct sequences of CBR4 were chosen, CBR4_1 and CBR4_2, and complexed with RNAiMAX, respectively (Figures S2A and S2D). Then, we used the rate constants that we obtained from modeling the kinetics of the SSB-RNAiMAX complex to fit the CBR4 in vitro data obtained above. As expected, we had to vary k4 (to account for different Ago2-binding kinetics for CBR4), while keeping all the other rate constants the same, to fit the actual CBR4-RNAiMAX data (Figures S2A and S2D). This provided us with a value of k4 for both CBR4_1 and CBR4_2, respectively. To our satisfaction, when we incorporated this k4 value with the other rate constants obtained for LNP05 and LNP(1,3)-diether (from the respective SSB modeling results), we were able to predict the kinetics of CBR4-LNP05 and CBR4-LNP(1,3)-diether (as illustrated in Figures S2B, S2C, S2E, and S2F, respectively, which shows the simulations plotted against the actual experimental results) in vitro. We also conducted preliminary in vivo studies with the above CBR4 sequences, encapsulated in LNP05 and LNP(1,3)-diether, respectively. The difference in activity predicted in vitro was also reflected in vivo (Figure S3).

## Discussion

Mathematical modeling, coupled with simple kinetic assays, provides an efficient way to probe the slow steps in the delivery of LNPs encapsulating an siRNA. Herein, we have described a mathematical model that is able to simulate the rates of different steps involved in the LNP-mediated delivery of siRNA in vitro. Although variations in network topology were explored in the course of model assembly, only the final configuration is described in the paper. This model represents a compact description of the intracellular RNAi pathway, achieved by focusing only on the critical steps involved in delivery. The kinetic model of siRNA-LNP complexes proposed in the present work consists of only five differential equations containing just nine parameters. Six of these parameters were fixed and independently determined (from literature or in-house experiments). The three remaining parameters (k1, k2, and k4), which indeed have the most impact on the optimization of siRNA/LNPs, were estimated from experimental data using a parameter-fitting technique. To the best of our knowledge, this is the simplest kinetic model that accurately replicates the behavior of LNPs in vitro. This model can be viewed as a simplification of Davis’ model, which was described by a system of 13 nonlinear equations containing 29 parameters. Reducing the number of differential equations and parameters makes our model more efficient from a computational point of view, and makes it more amenable to numerical simulation and analysis using readily available software (Simbiology platform from Mathworks).

The punctate pattern of intracellular siRNA-LNP complexes, as observed using confocal imaging (Figure 2A), suggests that LNPs become localized in intracellular vesicular compartments, possibly endosomes,^29^ highlighting endosomal escape as a potential key rate-limiting step in LNP-mediated delivery. As expected, our sensitivity analysis also showed that the endosomal escape/unpackaging parameter had the most significant impact on the model response, further validating the accuracy of our model (Figure 6). In fact, Gilleron et al.^30^ have also demonstrated that endosomal escape of siRNAs occurs at a very low efficiency and is indeed the rate-limiting step in delivery through elegant image-based analysis. We also observed a substantial delay in the onset of gene silencing for the two LNPs compared to RNAiMAX. This is consistent with published reports hypothesizing different mechanisms of cell uptake for LNPs and conventional transfection reagents, for example, non-endosomal routes and direct fusion with the cell membrane.^26^ Differential scanning calorimetry and encapsulation experiments performed in house also suggested that the siRNA cargo is loosely associated with RNAiMAX. This is in contrast to the LNP particles, where the siRNA is encapsulated within an ordered structure, suggesting that differential packaging of siRNA within the delivery vehicle could also contribute to differential knockdown kinetics. One limitation of the simple model described here is that by lumping endosomal escape and siRNA release into a single apparent rate constant, it is not possible to differentiate between these mechanisms with the available data. But as emphasized earlier, this first version of the model is currently developed and optimized for screening LNPs and siRNAs in vitro, and thus lumping endosomal escape and siRNA release into a single rate constant was not a concern for us.

We also observed that faster mRNA knock-down kinetics of LNP(1,3)-diether versus LNP05 translated into LNP(1,3)-diether being more potent than LNP05 in vitro and in vivo (Figures 3 and 5). The lipid composition used in the chosen LNPs was selected independently from a series of experiments based on the stability of the LNPs after assembly, their morphology, and their rigorous characterization using various analytical tools.^8^ Assuming an endosomal uptake mechanism for the LNP, the difference in activity of different classes of LNPs, as predicted by the model, may be attributed to different rates of endosomal escape. Imaging studies by Gilleron et al.^30^ have suggested that interactions of the cationic lipids with the endosomal membrane can affect the endosomal escape rates of siRNA cargo. In fact, 3D laser scanning confocal microscopy has revealed distinct interactions between LNPs, for both lamellar (L_α_) and inverted hexagonal (H_11_) nanostructures, and mouse fibroblast cells.^31^ Confocal imaging also showed that H_11_ complexes appear to rapidly fuse with cell membranes, resulting in higher transfection efficiency compared to L_α_ nanostructures. Preliminary in-house X-ray scattering experiments with endosomal membrane-mimicking lipids have shown that the phase transition temperature of the (1,3)-diether lipid is significantly lower than that of the Octyl ClinDMA, the cationic lipid used in LNP05. This could explain the propensity with which the former can transition from a lamellar phase to an inverted hexagonal phase and thereby fuse more readily with the membrane, aiding in a more efficient release of siRNA from the endosome and translating into higher potency both in vitro and in vivo. But we would like to again emphasize here that our goal was to be able to use this model for screening various siRNA-carrying vehicles for their relative efficacies, without getting into the mechanistic differences at this point. This approach can save time and effort that otherwise goes in conducting preliminary in vitro DOE studies for the selection of the most efficacious LNPs.

We have also demonstrated that the model can be successfully used to predict knockdown resulting from novel combinations of siRNAs and LNPs in vitro. The optimization of both the siRNA cargo and LNP vehicle can therefore be pursued independently, and application of the model can eliminate the need for redundant experimentation once the key parameters (k1, k2, and k4) are determined, thereby leading to a more efficient and less expensive approach of screening novel combinations of siRNAs and LNPs. We demonstrate the use of an in vitro Ago2-binding assay as a screening tool. Among the siRNA-LNP combinations that we tested in this study, the ones that had the fastest Ago2-binding kinetics in vitro produced the best knockdown of the respective target mRNA in vivo. We speculate that this trend should hold true upon testing additional siRNAs, but we would need a bigger dataset to make this conclusive correlation. We have also demonstrated the application of this model as a mechanistic platform for understanding the kinetics of competing siRNAs in combinations (manuscript under preparation).

In conclusion, the simple modeling approach described here enables the simulation of key steps involved in LNP-mediated siRNA delivery to cells in vitro, enabling the streamlining of experimentation involved in siRNA/LNP optimization. The benefit of our modeling approach is that it eliminates the need for extensive experimentation. We demonstrate that this approach can be extended to various other siRNA sequences. To validate our in vitro predictions, we performed preliminary in vivo studies, which showed an improvement in vivo of more than 2-fold with our delivery vehicle (LNP(1,3)-diether versus LNP05). We have also demonstrated that this model can be extended to the optimization of siRNA sequences, as demonstrated in our CBR4 experiments. CBR4_2 showed a more than 2-fold improvement over CBR4_1. This difference was also reflected in vivo (Figure S3). This gave us confidence on the predictive power of the model, although we still realize that this model is good for accurately predicting in vitro activity and will have to be further built upon to capture the complexities of in vivo delivery. To our satisfaction, the mathematical model successfully made quantitative predictions of siRNA/LNP activity in vitro and subsequent qualitative correlations in vivo. We also observed that 1 hr after dosing, the majority of the siRNA in the liver got associated with hepatocytes and that endosomal escape and inefficient RISC loading were significant barriers for delivery.^32^ This model serves as a starting point and focuses on the critical barriers in delivery. It also serves as a platform for the future development of next-generation models capable of capturing the additional complexity of in vivo delivery.

This model can potentially be extended to other types of delivery vehicles by refitting relevant parameters. It also provides a starting point for building in the additional steps involved in siRNA delivery in vivo. We have shown a scalable approach to support the optimization of delivery vehicles for siRNA therapeutics. This is made possible through systematic manipulation of key control parameters that govern the dynamics of RNAi silencing. In combination with iterative design and measurement, model exploration allows the drug developer to directly impact the drug discovery process, which will ultimately lead to the realization of the great potential of siRNA therapeutics.

## Materials and Methods

### Design and Synthesis of siRNA Sequences

Chemically modified siRNAs against SSB and CBR4 were synthesized by Merck/Sirna Therapeutics using already published methods.^33^ The sequence and chemical modifications are illustrated in the Supplemental Information. The first column in the table denotes the passenger strand from the 5′ to 3′ end, and the second column is the guide strand from the 3′ to 5′ end. Abbreviations for the modifications are as follows: d (deoxy), flu (2′ fluoro), ome (2′-O-methyl), ribo (ribose), and iB (inverted abasic nucleotide). For Cy5-labeled SSB siRNA, Cy5 (Invitrogen) was attached at the 5′ end of the passenger strand of the SSB duplex.

### Preparation of siRNA-Lipid Nanoparticle Complexes LNP201, LNP05, and LNP(1,3)-Diether

LNP201 was composed of Butyl CLinDMA (2-{4-[(3b)-cholest-5-en-3-yloxy]-butoxy}-N,N-dimethyl-3-[(9Z,12Z)-octadeca-9,12-dien-1-yloxy]propan-1-amine), cholesterol, and PEG-DMG (monomethoxy(polyethyleneglycol)-1,2-dimyristoylglycerol) in a 50.3:44.3:5.4 molar ratio, respectively, whereas LNP05 was composed of Octyl CLinDMA (2-{8-[(3b)-cholest-5-en-3-yloxy]-octyl}-N,N-dimethyl-3-[(9Z,12Z)-octadeca-9,12-dien-1-yloxy]propan-1-amine), cholesterol, and PEG-DMG in a 60:38:2 molar ratio, respectively. LNP(1,3)-diether had the same basic composition as LNP05; the only difference was in the cationic lipid used. The lipid used was 1-{6-[(3b)-cholest-5-en-3-yloxy]-hexyl}-N,N-dimethyl-3-[(9Z)-octadeca-9-dien-1-yloxy]propan-1-amine. All the siRNA-LNPs were assembled as previously described.^34^

### Cell Culture

HeLa cells and Hepa 1-6 cells were purchased from ATCC. Cells were cultured at 37°C under a 5% CO_2_ atmosphere in DMEM (containing 4.5 g/L of glucose) supplemented with 10% fetal bovine serum (Thermo Fischer Scientific), 100 IU/mL of penicillin G, and 100 μg/mL of streptomycin sulfate. Unless otherwise specified, cells were seeded at a density of between 6 × 10^3^ and 1 × 10^4^ cells per well in a 96-well plate, depending on the experiment.

### In Vitro siRNA Transfection

Cells were seeded on day 0, and the next day, cells were treated with LNP-formulated siRNAs or were transfected with RNAiMAX (Invitrogen) per the manufacturer’s protocol. To determine siRNA association and mRNA reduction, media was removed, and cells were washed once with 1 X PBS. Finally, PBS was removed, and the cells were lysed with Cells-to-Ct solution (Ambion). Lysates were analyzed for siRNA uptake and mRNA knockdown, as described elsewhere.^35^

### Confocal Microscopy

Cells were seeded on day 0 on collagen-coated 96-well plates (MatriCal), and the next day, they were incubated with LNP201 encapsulating Cy5-labeled SSB siRNA. Cells were incubated for different time points (1, 3, 6, and 12 hr) in an environmentally controlled chamber at 37°C and 5% CO_2_. Cells were counterstained with Cell Tracker Blue cytoplasmic dye (Invitrogen) 30 min prior to imaging. Live cells were imaged at an excitation wavelength of 646 nm and an emission wavelength of 666 nm using an OPERA confocal microscope (Perkin Elmer) equipped with a SensiCam QE digital charge-coupled device camera (PCO Imaging) and OPERA CN/QEHS software (version 1.8.1) using a 40×/0.90NA or a 20×/0.70NA water objective, and eight image locations were captured per well. Image analysis was performed using a custom script developed in Acapella v2.0 software (PerkinElmer) for the analysis of sub-cellular regions of interest. The membrane region of each cell was approximated to be a ring, with the outer boundary of this ring at the parameter of the cell (as defined by Cell Tracker dye) and a thickness of 6.5 μm. The cytoplasm region of each cell was approximated to cover all the area inside of the membrane-region ring, and the Acapella spot-detection algorithm was applied to the Cy5 image channel to segment small vesicle-like “spot” objects found within the cytoplasm region. Cy5 intensity, spot count measurements, and other measurements were collected from these regions.

### Ago2-Binding Experiments

Hepa1-6 cells were plated at a density of 4.4 × 10^6^/10-cm plate. The next day, cells were transfected with 10 nM SSB siRNA complexed with RNAiMAX, LNP05, or LNP(1,3)-diether, respectively, as described above for the kinetic studies. At selected time points, cells were lysed with 0.5% Triton X-100 lysis buffer, and Ago2 was precipitated as described previously.^36^

### In Vivo Experiments

C57BL/6 mice were intravenously dosed at 3 mg/kg with SSB, CBR4_1, or CBR4_2 siRNAs in (1,3)-diether and LNP05 delivery vehicles, respectively. The livers were collected 5 days post-dose, and the knockdown of the SSB and CBR4 mRNAs was determined relative to GAPDH expression. The log 2-fold change was relative to the PBS-treated animals. All in vivo work was approved by an Institutional Animal Care and Use Committee and adhered to standards recommended by the Association for Assessment and Accreditation of Laboratory Animal Care, International.

### Sensitivity Analysis

We carried out a sensitivity analysis using the Simbiology toolbox in MATLAB to determine the critical parameters that control intracellular siRNA delivery. The software computed local sensitivities by combining the original ODE system for a model with the auxiliary differential equations for the sensitivities.^37^ The solver solved this larger system of ODEs simultaneously. This was a straightforward and informative means to determine which parameter of the model had the greatest impact on a particular output or a particular model feature. This approach yielded accurate results.

## Author Contributions

R.M., E.K., B.G., and R.B. performed the experiments. R.M., E.C., D.R., D.B., and B.H. designed the experiments and analyzed and interpreted data. R.M., D.R., S.C., and J.J.C. wrote the manuscript.

## Supplemental Information

Document S1. Supplemental Materials and Methods and Figures S1–S3

Document S2. Article plus Supplemental Information
