# Supplementary material for: Mathematical Modeling: A Tool for Optimization of Lipid Nanoparticle-Mediated Delivery of siRNA
Source: Mol Ther Nucleic Acids. 2017 Apr 12;7:246–55. doi: 10.1016/j.omtn.2017.04.003 (PMC5415968; doi:10.1016/j.omtn.2017.04.003)
Supplement: Document S1. Supplemental Materials and Methods and Figures S1–S3 [file mmc1.pdf]

OMTN, Volume 7

Supplemental Information

Mathematical Modeling: A Tool for Optimization of Lipid Nanoparticle-Mediated Delivery of siRNA

Radu Mihaila, Dipali Ruhela, Edward Keough, Elena Cherkaev, Silvia Chang, Beverly Galinski, René Bartz, Duncan Brown, Bonnie Howell, and James J. Cunningham

SUPPORTING INFORMATION

FIGURE 1: Sequence and chemical modification information for the various siRNAs used in the study

	**Passenger Strand**	**Guide Strand**
**Cbr4_1**	iB;dG;dA;dG;dG;fluC;dA;dG;fluU;fluC;fluU;fluU;fluC;fluU;dA;dG;fluU;dA;dA;dG;dT;dT;iB	rC;rU;rU;omeA;fluC;fluU;omeA;omeG;omeA;omeA;omeG;omeA;fluC;fluU;omeG;fluC;fluC;fluU;fluC;omeU;omeU
**Cbr4_2**	iB;dG;dA;dA;dG;fluU;dA;fluU;fluU;dA;fluU;fluU;dG;dG;fluU;fluU;fluU;dG;dA;dA;dT;dT;iB	rU;rU;rC;omeA;omeA;omeA;fluC;fluC;omeA;omeA;fluU;omeA;omeA;fluU;omeA;fluC;fluU;fluU;fluC;omeU;omeU
**Ssb**	iB;dA;fluC;dA;dA;fluC;dA;dG;dA;fluC;fluU;fluU;fluU;dA;dA;fluU;dG;fluU;dA;dA;dT;dT;iB	rU;rU;rA;fluC;omeA;fluU;fluU;omeA;omeA;omeA;omeG;fluU;fluC;fluU;omeG;fluU;fluU;omeG;fluU;omeU;omeU

FIGURE 2

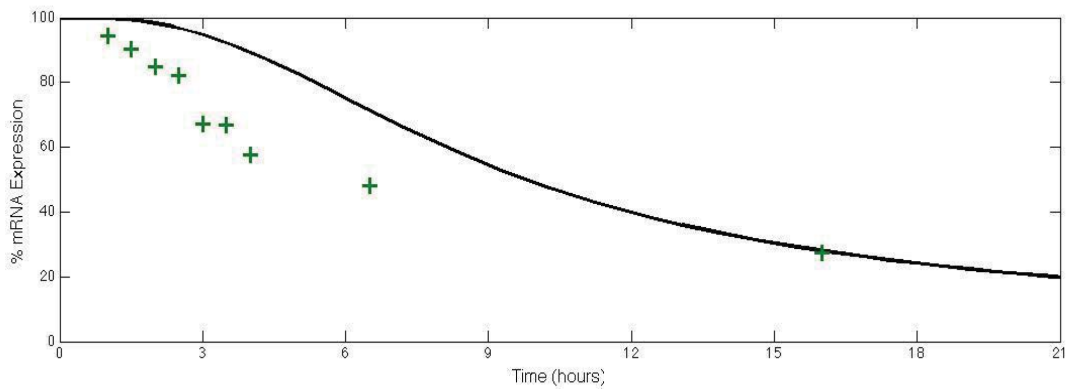

A

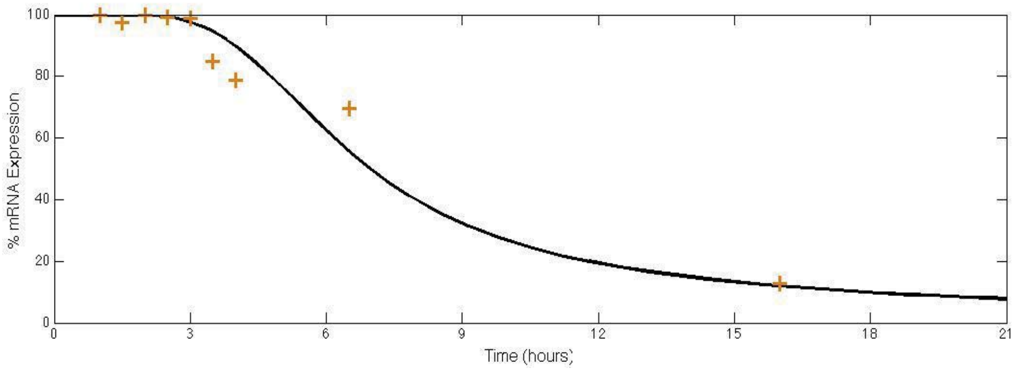
% mRNA Expression
Time (hours)

B

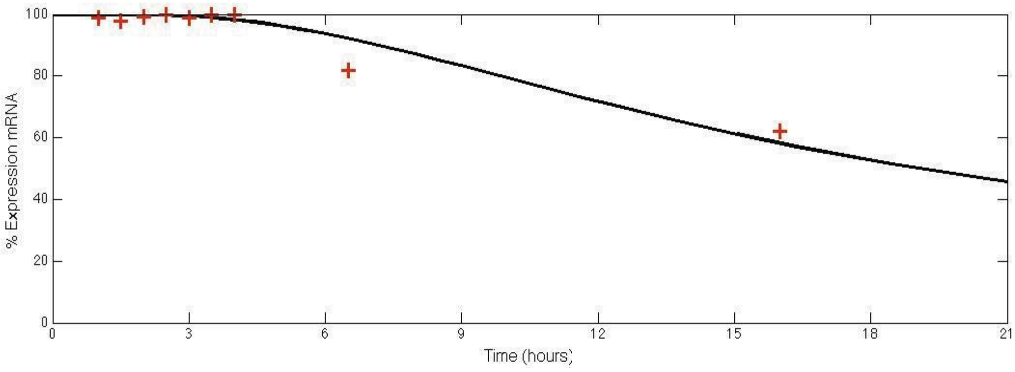
% Expression mRNA
Time (hours)

C

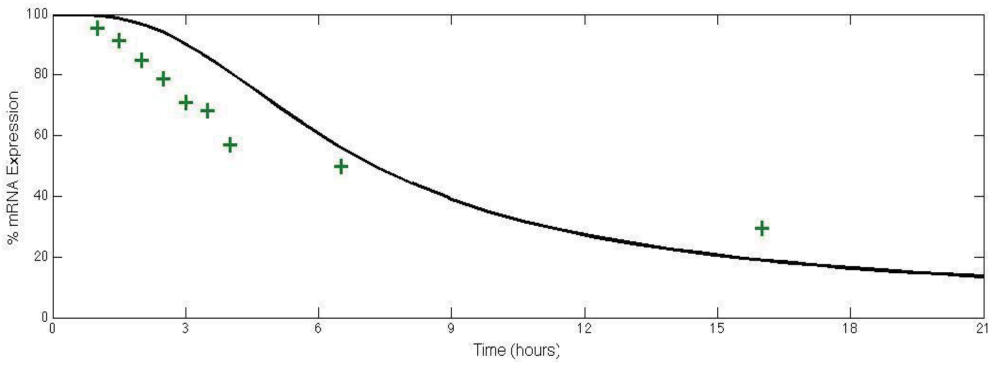
% mRNA Expression
Time (hours)

D

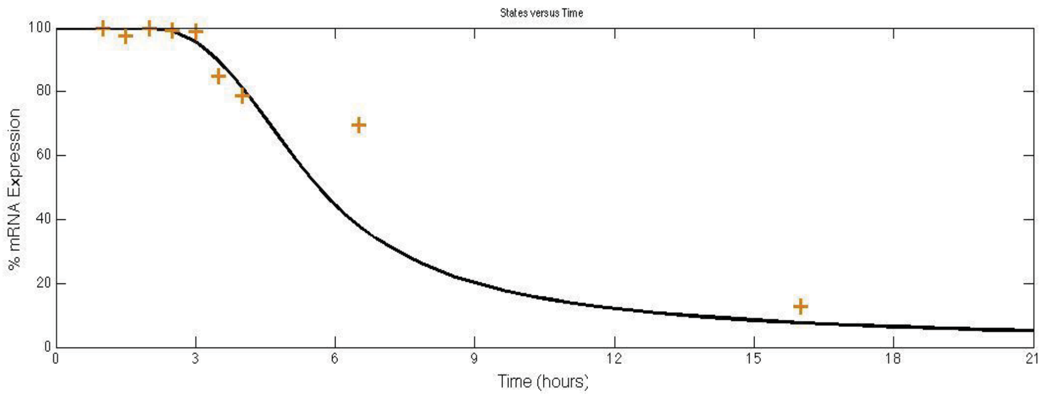

E

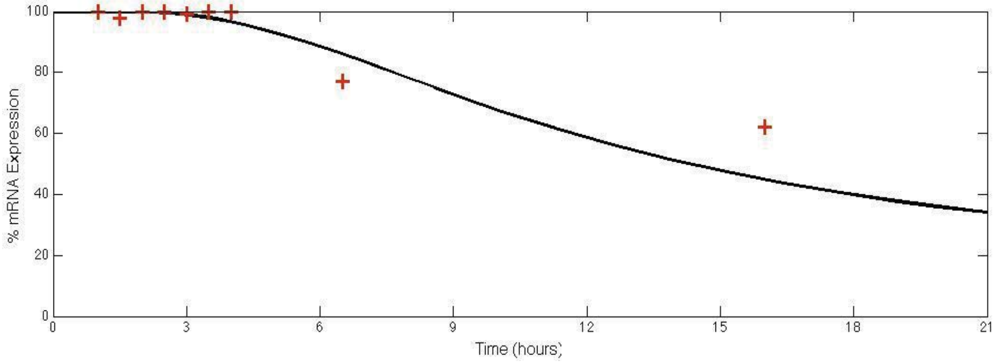

F

Quantitation of mRNA knock down with time for Cbr4_1 using **A**) RNAiMAX (green), **B**) LNP-(1,3)-diether (orange) and **C**) LNP05 (red) and for Cbr4_2 using **D**) RNAiMAX (green), **E**) LNP-(1,3)-diether (orange) and **F**) LNP05 (red) respectively at a concentration of 10 nM. Hepa1-6 cells at 10,000 cells per well were used for each transfection. Each data point is a mean of ten independent runs, with a cv% less than 5%. Statistical calculations were performed using Prism GraphPad software. The corresponding predictions from the mathematical model, using the baseline values as illustrated in Table 1, are shown in black curves in each plot.

FIGURE 3

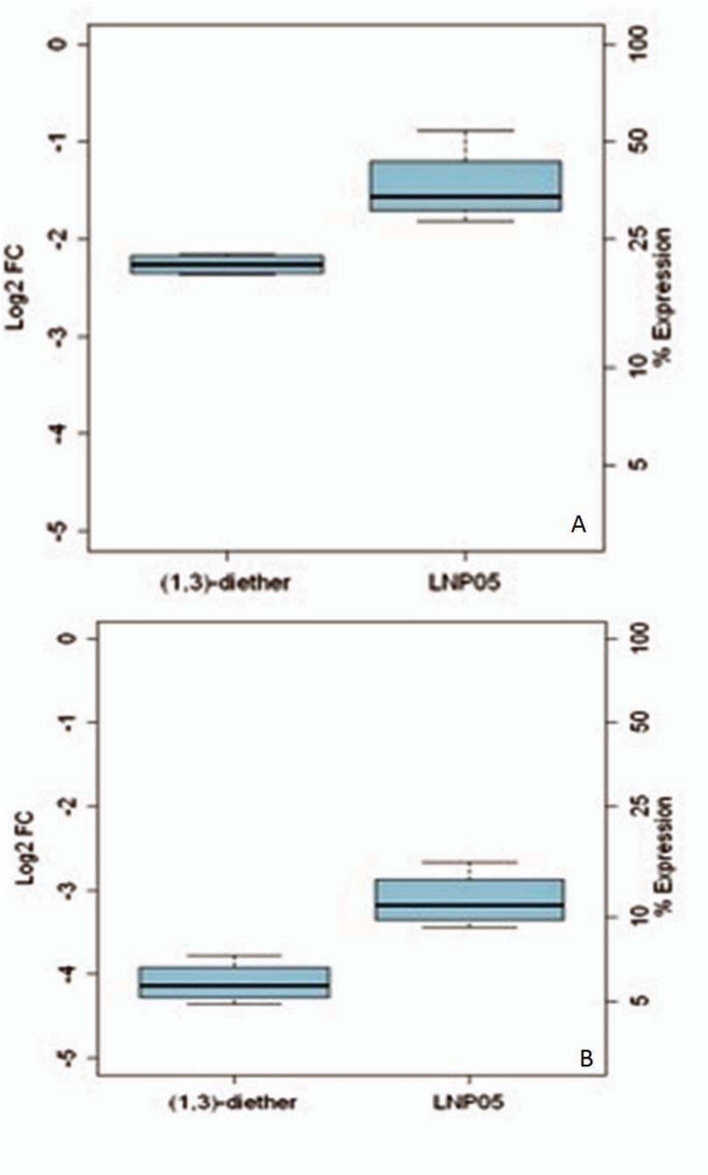

Preliminary evaluation of Cbr4_1 (**A**) and Cbr4_2 (**B**) gene silencing *in vivo* using LNP05 and (1,3)-diether vehicles on day 5. C57BL/6 mice were intravenously dosed at 3 mg/kg with (**A**) Cbr4_1 siRNA (**B**) Cbr4_2 siRNA in (1,3)-diether and LNP05 delivery vehicles respectively. The livers were collected 5 days post-dose and the knockdown of the Cbr4 mRNA

was determined relative to GAPDH expression. The log 2 fold change is relative to the PBS treated animals. There were 4 animals per treatment group and the error bars are ± SD.

MATLAB CODE:

```
<?xml version="1.0" encoding="UTF-8"?>
<sbml xmlns="http://www.sbml.org/sbml/level2/version4" level="2"
version="4">
  <annotation>
    <SimBiology xmlns="http://www.mathworks.com">
      <Version Major="5" Minor="5" Point="0"/>
    </SimBiology>
  </annotation>
  <model id="mw9a88fb4f_6d61_4ff9_9446_2de33fabf83e" name="Sirna Delivery
model_mtna">
    <listOfUnitDefinitions>
      <unitDefinition id="MWBUILTINUNIT_liter" name="liter">
        <listOfUnits>
          <unit kind="metre" exponent="3"/>
          <unit kind="dimensionless" multiplier="0.001"/>
        </listOfUnits>
      </unitDefinition>
      <unitDefinition id="MWBUILTINUNIT_molecule" name="molecule">
        <listOfUnits>
          <unit kind="mole" exponent="1" multiplier="1.66053872801495e-
24"/>
        </listOfUnits>
      </unitDefinition>
      <unitDefinition id="MWDERIVEDUNIT_1__molecule_1__hour"
name="(1/molecule)*(1/hour)">
        <listOfUnits>
          <unit kind="mole" exponent="-1"/>
          <unit kind="second" exponent="-1"/>
          <unit kind="dimensionless" multiplier="1.67281721944444e+20"/>
        </listOfUnits>
      </unitDefinition>
      <unitDefinition id="MWDERIVEDUNIT_1__hour" name="1/hour">
        <listOfUnits>
          <unit kind="second" exponent="-1"/>
          <unit kind="dimensionless" multiplier="0.000277777777777778"/>
        </listOfUnits>
      </unitDefinition>
      <unitDefinition id="MWDERIVEDUNIT_molecule__hour"
name="molecule/hour">
        <listOfUnits>
          <unit kind="mole" exponent="1"/>
          <unit kind="second" exponent="-1"/>
          <unit kind="dimensionless" multiplier="4.6126075778193e-28"/>
        </listOfUnits>
      </unitDefinition>
```

```
    </listOfUnitDefinitions>
    <listOfCompartments>
      <compartment id="mwcdd6ab06_8d77_43f1_9bd7_38537dd32e8c"
name="Extracellular" spatialDimensions="3" size="0.0003"
units="MWBUILTINUNIT_liter" constant="true"/>
      <compartment id="mwf856c93b_a8ce_4f69_ab16_1199345e26c8"
name="Intracellular" spatialDimensions="3" size="1.4e-12"
units="MWBUILTINUNIT_liter" constant="true"/>
    </listOfCompartments>
    <listOfSpecies>
      <species id="mwccc1d589_268a_4a53_91a5_6c61e11561bc" name="E"
compartment="mwcdd6ab06_8d77_43f1_9bd7_38537dd32e8c"
initialAmount="30000000" substanceUnits="MWBUILTINUNIT_molecule"
hasOnlySubstanceUnits="true" boundaryCondition="false" constant="false"/>
      <species id="mw59a4a09c_1b15_488f_a68d_3111ca1ea134" name="S"
compartment="mwf856c93b_a8ce_4f69_ab16_1199345e26c8" initialAmount="0"
substanceUnits="MWBUILTINUNIT_molecule" hasOnlySubstanceUnits="true"
boundaryCondition="false" constant="false"/>
      <species id="mw300ef6a7_017b_491b_a4fc_22434960410c" name="R"
compartment="mwf856c93b_a8ce_4f69_ab16_1199345e26c8" initialAmount="10000"
substanceUnits="MWBUILTINUNIT_molecule" hasOnlySubstanceUnits="true"
boundaryCondition="false" constant="false"/>
      <species id="mwbf9f846e_9c75_4d54_b1d7_232eeacd9df6" name="SR"
compartment="mwf856c93b_a8ce_4f69_ab16_1199345e26c8" initialAmount="0"
substanceUnits="MWBUILTINUNIT_molecule" hasOnlySubstanceUnits="true"
boundaryCondition="false" constant="false"/>
      <species id="mwb6bfb6cf_48b0_407e_a4f6_9f306864faca" name="SRM"
compartment="mwf856c93b_a8ce_4f69_ab16_1199345e26c8" initialAmount="0"
substanceUnits="MWBUILTINUNIT_molecule" hasOnlySubstanceUnits="true"
boundaryCondition="false" constant="false"/>
      <species id="mwc7da845e_cc60_4265_a108_d00b5442e2d4" name="M"
compartment="mwf856c93b_a8ce_4f69_ab16_1199345e26c8" initialAmount="100"
substanceUnits="MWBUILTINUNIT_molecule" hasOnlySubstanceUnits="true"
boundaryCondition="false" constant="false"/>
      <species id="mw81a9608a_50d7_4e6b_8f05_272707e76da7" name="N"
compartment="mwf856c93b_a8ce_4f69_ab16_1199345e26c8" initialAmount="0"
substanceUnits="MWBUILTINUNIT_molecule" hasOnlySubstanceUnits="true"
boundaryCondition="false" constant="false"/>
    </listOfSpecies>
    <listOfReactions>
      <reaction id="mw8b0e8b42_d753_455a_a8cd_4fc78d83e876" name="siRNA
Ago2 loading" reversible="false" fast="false">
        <listOfReactants>
          <speciesReference
species="mw300ef6a7_017b_491b_a4fc_22434960410c" stoichiometry="1"/>
          <speciesReference
species="mw59a4a09c_1b15_488f_a68d_3111ca1ea134" stoichiometry="1"/>
        </listOfReactants>
        <listOfProducts>
          <speciesReference
species="mwbf9f846e_9c75_4d54_b1d7_232eeacd9df6" stoichiometry="1"/>
        </listOfProducts>
        <kineticLaw>
          <math xmlns="http://www.w3.org/1998/Math/MathML">
```

```
            <apply>
              <times/>
              <ci> mwe19297b0_02f2_401d_9cb2_07044dbb3ef4 </ci>
              <ci> mw300ef6a7_017b_491b_a4fc_22434960410c </ci>
              <ci> mw59a4a09c_1b15_488f_a68d_3111ca1ea134 </ci>
            </apply>
          </math>
          <listOfParameters>
            <parameter id="mwe19297b0_02f2_401d_9cb2_07044dbb3ef4"
name="k4" value="0.001" units="MWDERIVEDUNIT_1__molecule_1__hour"
constant="true"/>
          </listOfParameters>
        </kineticLaw>
      </reaction>
      <reaction id="mw1637383a_98fc_4e2f_9105_83d2dcd2b2c6"
name="Formation Active RISC-mRNA complex" reversible="false" fast="false">
        <listOfReactants>
          <speciesReference
species="mwbf9f846e_9c75_4d54_b1d7_232eeacd9df6" stoichiometry="1"/>
          <speciesReference
species="mwc7da845e_cc60_4265_a108_d00b5442e2d4" stoichiometry="1"/>
        </listOfReactants>
        <listOfProducts>
          <speciesReference
species="mwb6bfb6cf_48b0_407e_a4f6_9f306864faca" stoichiometry="1"/>
        </listOfProducts>
        <kineticLaw>
          <math xmlns="http://www.w3.org/1998/Math/MathML">
            <apply>
              <times/>
              <ci> mwad8371fb_b325_4fb4_a538_ed51253ee7db </ci>
              <ci> mwbf9f846e_9c75_4d54_b1d7_232eeacd9df6 </ci>
              <ci> mwc7da845e_cc60_4265_a108_d00b5442e2d4 </ci>
            </apply>
          </math>
          <listOfParameters>
            <parameter id="mwad8371fb_b325_4fb4_a538_ed51253ee7db"
name="k6" value="0.1" units="MWDERIVEDUNIT_1__molecule_1__hour"
constant="true"/>
          </listOfParameters>
        </kineticLaw>
      </reaction>
      <reaction id="mwc2ff4eea_d1f4_41a8_9f71_649b4f2c687b" name="Active-
RISC-mRNA cleavage" reversible="false" fast="false">
        <listOfReactants>
          <speciesReference
species="mwb6bfb6cf_48b0_407e_a4f6_9f306864faca" stoichiometry="1"/>
        </listOfReactants>
        <listOfProducts>
          <speciesReference
species="mwbf9f846e_9c75_4d54_b1d7_232eeacd9df6" stoichiometry="1"/>
        </listOfProducts>
        <kineticLaw>
          <math xmlns="http://www.w3.org/1998/Math/MathML">
```

```
            <apply>
              <times/>
              <ci> mwd850ad36_f79f_49cb_b107_1a28998082c0 </ci>
              <ci> mwb6bfb6cf_48b0_407e_a4f6_9f306864faca </ci>
            </apply>
          </math>
          <listOfParameters>
            <parameter id="mwd850ad36_f79f_49cb_b107_1a28998082c0"
name="k7" value="7.2" units="MWDERIVEDUNIT_1__hour" constant="true"/>
          </listOfParameters>
        </kineticLaw>
      </reaction>
      <reaction id="mw2e9b6ec9_552e_4631_9ee4_d0b6925bee59"
name="Endosomal LNP degradation" reversible="false" fast="false">
        <listOfReactants>
          <speciesReference
species="mw81a9608a_50d7_4e6b_8f05_272707e76da7" stoichiometry="1"/>
        </listOfReactants>
        <kineticLaw>
          <math xmlns="http://www.w3.org/1998/Math/MathML">
            <apply>
              <times/>
              <ci> mwb1db6c2d_ad9a_4916_9d9d_06b180f4cbda </ci>
              <ci> mw81a9608a_50d7_4e6b_8f05_272707e76da7 </ci>
            </apply>
          </math>
          <listOfParameters>
            <parameter id="mwb1db6c2d_ad9a_4916_9d9d_06b180f4cbda"
name="k3" value="3" units="MWDERIVEDUNIT_1__hour" constant="true"/>
          </listOfParameters>
        </kineticLaw>
      </reaction>
      <reaction id="mw835b4e93_613f_4d28_a226_e2e02dd6d047"
name="transcription" reversible="false" fast="false">
        <listOfProducts>
          <speciesReference
species="mwc7da845e_cc60_4265_a108_d00b5442e2d4" stoichiometry="1"/>
        </listOfProducts>
        <kineticLaw>
          <math xmlns="http://www.w3.org/1998/Math/MathML">
            <ci> mwe231d9d6_540b_4165_8ad1_57353940bd07 </ci>
          </math>
          <listOfParameters>
            <parameter id="mwe231d9d6_540b_4165_8ad1_57353940bd07"
name="k8" value="100" units="MWDERIVEDUNIT_molecule__hour"
constant="true"/>
          </listOfParameters>
        </kineticLaw>
      </reaction>
      <reaction id="mw6d92b1d5_f1aa_436e_bbd5_a82d189aa1a4"
name="degradation of mRNA" reversible="false" fast="false">
        <listOfReactants>
          <speciesReference
species="mwc7da845e_cc60_4265_a108_d00b5442e2d4" stoichiometry="1"/>
```

```
        </listOfReactants>
        <kineticLaw>
          <math xmlns="http://www.w3.org/1998/Math/MathML">
            <apply>
              <times/>
              <ci> mw42639391_24a6_4fb1_b8e9_cdecfb040e6d </ci>
              <ci> mwc7da845e_cc60_4265_a108_d00b5442e2d4 </ci>
            </apply>
          </math>
          <listOfParameters>
            <parameter id="mw42639391_24a6_4fb1_b8e9_cdecfb040e6d"
name="k9" value="1" units="MWDERIVEDUNIT_1__hour" constant="true"/>
          </listOfParameters>
        </kineticLaw>
      </reaction>
      <reaction id="mwa7b8c7e4_eb8f_48fa_bcbf_f3e324c5ebac"
name="degradation free sirna" reversible="false" fast="false">
        <listOfReactants>
          <speciesReference
species="mw59a4a09c_1b15_488f_a68d_3111ca1ea134" stoichiometry="1"/>
        </listOfReactants>
        <kineticLaw>
          <math xmlns="http://www.w3.org/1998/Math/MathML">
            <apply>
              <times/>
              <ci> mw48c5d376_6cb1_446c_9e91_ff7c013b09c4 </ci>
              <ci> mw59a4a09c_1b15_488f_a68d_3111ca1ea134 </ci>
            </apply>
          </math>
          <listOfParameters>
            <parameter id="mw48c5d376_6cb1_446c_9e91_ff7c013b09c4"
name="k5" value="0.03" units="MWDERIVEDUNIT_1__hour" constant="true"/>
          </listOfParameters>
        </kineticLaw>
      </reaction>
      <reaction id="mw37a57427_559f_46d7_8be2_d39b8743f23a" name="crossing
of cell membrane into endosome" reversible="false" fast="false">
        <listOfReactants>
          <speciesReference
species="mwccc1d589_268a_4a53_91a5_6c61e11561bc" stoichiometry="1"/>
        </listOfReactants>
        <listOfProducts>
          <speciesReference
species="mw81a9608a_50d7_4e6b_8f05_272707e76da7" stoichiometry="1"/>
        </listOfProducts>
        <kineticLaw>
          <math xmlns="http://www.w3.org/1998/Math/MathML">
            <apply>
              <times/>
              <ci> mw2800d0d1_f97b_406d_af13_bf8f9c63354e </ci>
              <ci> mwccc1d589_268a_4a53_91a5_6c61e11561bc </ci>
            </apply>
          </math>
          <listOfParameters>
```

```
            &lt;parameter id="mw2800d0d1_f97b_406d_af13_bf8f9c63354e"
name="k1" value="0.005" units="MWDERIVEDUNIT_1__hour" constant="true"/>
          &lt;/listOfParameters&gt;
        &lt;/kineticLaw&gt;
      &lt;/reaction&gt;
      <reaction id="mwbadc16bb_e225_489e_b0e5_73a603b694a4"
name="endosomal escape" reversible="false" fast="false">
        &lt;listOfReactants&gt;
          <speciesReference
species="mw81a9608a_50d7_4e6b_8f05_272707e76da7" stoichiometry="1"/>
        &lt;/listOfReactants&gt;
        &lt;listOfProducts&gt;
          <speciesReference
species="mw59a4a09c_1b15_488f_a68d_3111ca1ea134" stoichiometry="1"/>
        &lt;/listOfProducts&gt;
        &lt;kineticLaw&gt;
          &lt;math xmlns="http://www.w3.org/1998/Math/MathML"&gt;
            &lt;apply&gt;
              &lt;times/&gt;
              &lt;ci&gt; mw1ff1db26_07c7_460c_a0d1_e183eb189d71 &lt;/ci&gt;
              &lt;ci&gt; mw81a9608a_50d7_4e6b_8f05_272707e76da7 &lt;/ci&gt;
            &lt;/apply&gt;
          &lt;/math&gt;
          &lt;listOfParameters&gt;
            &lt;parameter id="mw1ff1db26_07c7_460c_a0d1_e183eb189d71"
name="k2" value="0.0005" units="MWDERIVEDUNIT_1__hour" constant="true"/>
          &lt;/listOfParameters&gt;
        &lt;/kineticLaw&gt;
      &lt;/reaction&gt;
    &lt;/listOfReactions&gt;
  &lt;/model&gt;
&lt;/sbml&gt;
```
